# Compliance with Good Manufacturing Practice in the Assessment of Immunomodulation Potential of Clinical Grade Multipotent Mesenchymal Stromal Cells Derived from Wharton’s Jelly

**DOI:** 10.3390/cells8050484

**Published:** 2019-05-21

**Authors:** Marta Grau-Vorster, Luciano Rodríguez, Anna del Mazo-Barbara, Clémentine Mirabel, Margarita Blanco, Margarita Codinach, Susana G. Gómez, Sergi Querol, Joan García-López, Joaquim Vives

**Affiliations:** 1 Banc de Sang i Teixits, Edifici Dr. Frederic Duran i Jordà, Passeig Taulat, 116, 08005 Barcelona, Spain; mgrau@bst.cat (M.G.-V.); lrodriguez@bst.cat (L.R.); annaaa.dm@gmail.com (A.d.M.-B.); clementine.mirabel@gmail.com (C.M.); mblanco@bst.cat (M.B.); mcodinach@bst.cat (M.C.); gomez.susana@gmail.com (S.G.G.); squerol@bst.cat (S.Q.); 2 Transfusion Medicine Group, Vall d′Hebron Research Institute (VHIR), Universitat Autònoma de Barcelona, Passeig de la Vall d’Hebron 129-139, 08035 Barcelona, Spain; 3 Transfusion Medicine and Cellular and Tissue Therapies, Universitat Autònoma de Barcelona, Campus UAB, Cerdanyola del Vallès, 08035 Bellaterra, Spain; 4 Musculoskeletal Tissue Engineering Group, Vall d′Hebron Research Institute (VHIR), Universitat Autònoma de Barcelona, Passeig de la Vall d’Hebron 129-139, 08035 Barcelona, Spain; 5 Departament de Medicina, Universitat Autònoma de Barcelona, Passeig de la Vall d’Hebron 129-139, 08035 Barcelona, Spain

**Keywords:** multipotent mesenchymal stromal cell, immunomodulation, proliferation assay, cellular therapy, cell culture, good manufacturing practice, quality by design

## Abstract

**Background**: The selection of assays suitable for testing the potency of clinical grade multipotent mesenchymal stromal cell (MSC)-based products and its interpretation is a challenge for both developers and regulators. Here, we present a bioprocess design for the production of Wharton’s jelly (WJ)-derived MSCs and a validated immunopotency assay approved by the competent regulatory authority for batch release together with the study of failure modes in the bioprocess with potential impact on critical quality attributes (CQA) of the final product. **Methods**: The lymphocyte proliferation assay was used for determining the immunopotency of WJ-MSCs and validated under good manufacturing practices (GMP). Moreover, failure mode effects analysis (FMEA) was used to identify and quantify the potential impact of different unexpected situations on the CQA. **Results**: A production process based on a two-tiered cell banking strategy resulted in batches with sufficient numbers of cells for clinical use in compliance with approved specifications including MSC identity (expressing CD73, CD90, CD105, but not CD31, CD45, or HLA-DR). Remarkably, all batches showed high capacity to inhibit the proliferation of activated lymphocytes. Moreover, implementation of risk management tools led to an in-depth understanding of the manufacturing process as well as the identification of weak points to be reinforced. **Conclusions**: The bioprocess design showed here together with detailed risk management and the use of a robust method for immunomodulation potency testing allowed for the robust production of clinical-grade WJ-MSCs under pharmaceutical standards.

## 1. Introduction

Cell-based medicinal products hold the promise to bring therapeutic alternatives to address unmet medical needs [1,2,3]. Amongst the diverse cell types that have attracted clinical interest, multipotent mesenchymal stromal cells (MSC) emerge as strong candidates, with several clinical trials already completed demonstrating an excellent safety profile [4]. Nonetheless, solid data on efficacy are jeopardized by poor translation of preclinical results that may have been interpreted too optimistically [5,6]. In this sense, a huge effort is currently being made to define specific target conditions that could be realistically treated with such therapies, which requires robust methods of production and suitable potency assays directly related to the mechanism of action [7]. This is a major challenge for both developers and regulators, since the pharmacological activity of MSCs is poorly understood, and the choice of an appropriate potency assay needs to be agreed with the competent regulatory authority. Potency assays are defined as the “quantitative measure of biological activity based on the attribute of the product” according to the European Guideline on Human Cell-Based Medicinal Products, EMEA/CHMP/410869/2006. However, mechanisms of action are complex and not fully understood. Furthermore, other limitations such as variability in starting materials, limited stability, and lot size also increase the difficulty in establishing adequate potency tests as well as an accurate panel of product specifications.

In the context of MSC-based therapy for the treatment of immunological disorders, the potency assays most commonly used are based on the determination of their in vitro immunomodulation capacity [8]. Moreover, to further ensure the quality of medicines, we followed an approach known as quality by design (QbD), which is welcomed by the European Medicines Agency (EMA) and allows for the control of bioprocesses by employing statistical, analytical, and risk-management methodology. According to quality risk management, we studied in detail potential failure risks in critical steps involved in the production process using failure mode and effects analysis (FMEA) [9] and identified parameters that may lead to potential out-of-specification (OOS) of any of the critical quality attributes (CQA) of the final Wharton jelly (WJ) MSC-based product, with special attention being given to the immunopotency assay currently used for batch release.

Here, we present a validated production process compliant with current good manufacturing practice (GMP) that addresses both (a) the challenge of establishing a robust bioprocess design and (b) defining appropriate quality controls (QC), which include meaningful potency assays.

## 2. Materials and Methods

### 2.1. Source Tissue, MSC Derivation, and Expansion

WJ-MSCs were derived following GMP-compliant procedures reported elsewhere [10], within the context of a clinical trial (EudraCT No. 2015-005786-23) with appropriate donor informed consent. Briefly, a fragment of umbilical cord tissue compliant with acceptance criteria described in Table 1 was cut longitudinally and split open so that the two arteries and the vein could be removed by pulling them gently. WJ was scrapped with a surgical scalpel, spread uniformly over the plastic surface of a T-flask with re-closable lid (TPP), and incubated for 30 min at 37 °C. After the incubation, 10 mL of Dulbecco’s modified Eagle’s medium (DMEM; Gibco, Carlsbad, CA, USA) containing 2 mmol/L glutamine was added and supplemented with 2 × 10^4^ UI/mL penicillin (Invitrogen, New York, NY, USA), 20 mg/mL streptomycin (Invitrogen), 120 μg/mL amphotericin B (Invitrogen), and 20% human serum B (hSerB, Banc de Sang i Teixits, Barcelona, Spain). After 2–5 days, a washing step with saline solution was performed, and 10 mL of fresh derivation medium was added. From this point, the culture medium was replaced every 3–4 days. Cells were further expanded in vitro by seeding cell culture flasks at (1–3) × 10^3^ cell/cm^2^ in derivation medium. When the total number of cells reached at least 5 × 10^6^, they were frozen in cryovials producing the master cell bank (MCB). Further expansion was performed after thawing for the generation of either working cell bank (WCB) or drug product (DP) directly using expansion medium composed of DMEM containing 2 mmol/L glutamine and supplemented with 10% hSerB (that is, “expansion medium”). Final product (FP) was defined as the product resulting from thawing a DP, washed and conditioned for administration in patients, which complies with criteria presented in Table 2. All cultures were maintained at 37 °C and 5% CO_2_ in humidified incubators. Media were replaced every 3–4 days, and trypsinization was performed at 70–90% confluence.

### 2.2. Flow Cytometry

Cells were numbered using Perfect-Count MicrospheresTM (Cytognos, Salamanca, Spain) microbeads in a FACSCalibur cytometer (Becton Dickinson, San Jose, CA, USA). Viability was determined using the 7-Amino-Actinomycin D (7-AAD, BD Biosciences, San Jose, CA, USA) exclusion method. Data were analyzed with CellQuest Pro (Becton Dickinson) software. In accordance with the ISCT criteria [11], mesenchymal identity was evaluated by the expression of surface markers CD31 (clone WM59; BD Biosciences), CD45 (clone HI30; BD Biosciences), CD73 (clone AD2; BD Biosciences), CD90 (clone F15-42-1-5; Beckman Coulter Inc, Miami, FL, USA), CD105 (clone 43A4E1; Miltenyi Biotec, Bergish Gladbach, Germany), and HLA-DR (clone L243; BD Biosciences) in a FACSCalibur device. PE- and FITC-conjugated IgG1k (G18-145, BD Biosciences) antibodies were used as isotype controls. Cells were stained for 15 min at room temperature, washed, and resuspended with PBS (Invitrogen). Acquisition was done using a FACSCalibur, and data were analyzed with CellQuest Pro software (version 5.2.1, BD Biosciences).

### 2.3. Lymphocyte Proliferation Assays

The immunomodulation potential of WJ-MSCs was determined by their capacity to inhibit the proliferation of polyclonally stimulated lymphocytes in vitro, as comprehensively described elsewhere [12]. MNC were obtained by density gradient (Histopaque-1077; Sigma-Aldrich, Saint Louis, MO, USA) from 24- to 48-hour-old buffy-coats or peripheral blood of healthy blood donors, which were confirmed negative for hepatitis B virus (HBV), hepatitis C virus (HCV), human immunodeficiency virus (HIV), and syphilis, both by serology and viral nucleic acid detection (NAD). Next, 2.5 × 10^6^ MNC/mL were labelled with 0.625 µM carboxy–fluorescein diacetate succinimidyl ester (CFSE) for 10 min using the CellTrace™ CFSE Cell Proliferation Kit (Molecular Probes, Eugene, OR, USA). After washing, (1–2) × 10^7^ cells/mL were incubated for 12 min at 37 °C, washed again and seeded onto flat-bottomed 96-well plates (Corning, Corning, NY, USA) at an MNC:WJ-MSC 5:1 ratio. Activation of lymphocytes was done with 25 ng/mL Phorbol 12-myristate 13-acetate (PMA, Sigma-Aldrich) and 0.5 µM Ionomycin (Sigma-Aldrich) in a final volume of 0.5 mL/well of “expansion medium”. Proliferation of MNC was determined by measuring the reduction of fluorescence intensity at day 5 using flow cytometry (FACSCalibur, Becton Dickinson), and data were analyzed with the FlowJo software (TreeStar Inc., Ashland, OR, USA).

### 2.4. Karyotype

Metaphase chromosome spreads (≥20 per sample) were prepared from each cell line in the exponential phase of growth as reported elsewhere [13]. Briefly, 25 µL of colcemid (Life Technologies, Carlsbad, CA, USA) were added to the cultures before trypsinization for arresting the cells in metaphase. After trypsinization, cells were fixed with Carnoy. Wrigth’s stain was used to stain chromosomes. Then, metaphase spreads were captured and karyotyped using an automated imaging system for cytogenetics (Cytovision, Applied Imaging, Sunderland, UK).

### 2.5. Risk Analysis

Quality risk management, defined by the World Health Organization (WHO) as a systematic process for the assessment, control, communication and review of risks to the quality of the medicinal product, was performed here in accordance with the recommendations of the International Council for Harmonization (ICH) guideline Q9 on Quality Risk Management and WHO Good manufacturing practices for pharmaceutical products Annex 2 guidelines. Two different steps were followed:

#### 2.5.1. Risk Assessment

Risk assessment involved identification, analysis of probability and severity, and evaluation of the level and need to mitigate potential risks (Table 3). After risk identification, the quantification of the potential risks was done by performing a failure mode effect analysis (FMEA) [9,14]. This is a quantitative tool permitting to evaluate potential risk situations, causes, and consequences, and the evaluation of suitable corrective actions by means of the risk priority number (RPN) that takes into consideration the occurrence of the potential risk, the possible severity, and the probability of detecting the failure (Equation (1)). Along the risk evaluation, the severity effects and the likelihood of the occurrence were assigned to a score from 1 to 5 (Tables S1 and S2). The likelihood that potential failure modes are detected before altering the product quality was rated by detection values (Table S3). Failure groups are listed in (Table S4).

(1) RPN=Severity×Occurence×Detection

#### 2.5.2. Risk Control

Risk control involved risk reduction or risk acceptance. Internally, RPN was classified in three grades, low (RPN < 20), medium (RPN 20–55), and high (RPN > 55) vulnerability. Corrective actions were implemented for those failures with an RPN number higher than the aim to minimize the occurrence and/or enhance its detectability. Risk evaluation was carried out again after corrective actions.

#### 2.5.3. Graphical Representation

Provided that it is unlikely to perform all tests resulting from the FMEA, we suggest plotting a Pareto’s chart with the objective of identifying the risks that account for 80% of failures, which typically arise from 20% of the sources of risk [9].

### 2.6. Microbiological Testing

Absence of microbial contamination of MCB and WCB was verified by inoculation in iFA PLUS and iFN PLUS media bottles (Biomerieux Industries) and incubation at 37 °C for 14 days. Sterility tests of DP were conducted according to European Pharmacopea (EuPh). Anaerobic bacteria were cultured on fluid thioglycolate medium (Biomerieux, Marcy l′Etoile, France) at 30–35 °C. Fungi and aerobic bacteria were cultured on Soya-bean casein digest medium (Biomerieux) at 20–25 °C. Samples were incubated for at least 14 days and then inspected visually. If no evidence of microbial growth was found, the product was said to comply with the test for sterility. Additionally, Gram staining tests were used for parametric release of the finished product, provided that EuPh sterility tests take two weeks. Briefly, the test was conducted in duplicates for confirmatory purposes by overnight incubation at 37 °C using three different media, namely: Columbia agar supplemented with 5% sheep blood, Chocolate agar PolyViteX, and Sabouraud Gentamicin Chloramphenicol 2 agar (Biomerieux). If the result was negative, then the product was considered compliant.

### 2.7. Endotoxin, Mycoplasm and Adventitious Virus Tests

The Endosafe-PTS system (Charles River, Wilmington, MA, USA) was used following the manufacturer’s instructions. The Venor qEP (Minerva Biolabs, Berlin, Germany) Kit was used for the amplification of a mycoplasma-specific 16S rRNA gene region and detection by real time polymerase chain reaction (PCR) according to the manufacturer’s instructions. In vitro adventitious agents’ detection in MSC samples were performed on MRC5 and Vero cells. If cytopathic effect was observed after 14–28 days of culture, PCR and immunofluorescence techniques were used to identify contamination agents.

### 2.8. Statistical Analysis

Descriptive data were expressed as mean ± standard deviation (number).

## 3. Results

### 3.1. Bioprocess Design

**Figure 1:**
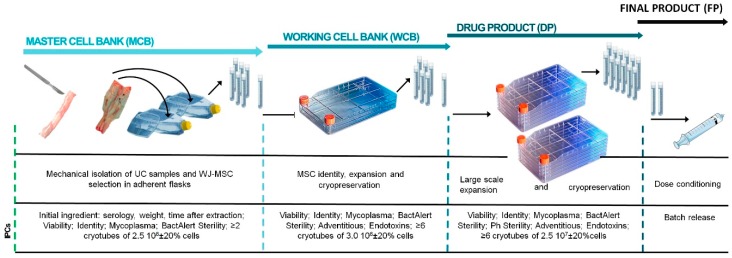
Schematic of bioprocess and established in-process controls (IPCs) for the follow up of the critical quality attributes for starting material and drug product. Although initial setup included an intermediate working cell bank, the competent regulatory authority allowed the possibility to expand drug product (DP) directly from the master cell bank (MCB). UC: Umbilical cord; WJ-MSC: Wharton jelly–mesenchymal stromal cells; IPC: In-process controls; Ph: Pharmacopea.

The bioprocess design proposed here allowed for the successful isolation of MSCs from the Wharton’s jelly harvested from umbilical cord tissue discarded after birth in maternities associated to our blood and tissue bank. Cells were subsequently expanded in vitro up to sufficient numbers following a GMP-compliant manufacturing process design presented in Figure 1 that involves the establishment of cryopreserved intermediate elements (namely, MCB, WCB, and DP) to obtain a cell-based product in a timely and cost-effective manner. The necessary steps for the establishment of the different intermediates and the generation of the FP (Final Product) were analyzed to identify the contamination risks, as detailed next:

#### 3.1.1. Master Cell Bank (MCB)

MCB involved the isolation, selection, expansion, and cryopreservation of WJ-MSCs. Through these steps, the following microbiological testing were performed: The presence of mycoplasma after cell expansion, a BactAlert sterility test before freezing, and a reference sample for endotoxin testing, in case abnormal levels of endotoxins were detected in the later phases.

#### 3.1.2. Working Cell Bank (WCB)

WCB involved the thawing of a cryopreserved unit of MCB, subsequent cell culture expansion, and cryopreservation. The presence of mycoplasma, adventitious virus, and endotoxins was evaluated after product concentration for cryopreservation. A BactAlert sterility test was also performed before freezing.

#### 3.1.3. Drug Product (DP)

DP was obtained following a similar procedure, in which a cryopreserved unit of WCB was thawed and cells were expanded and frozen. After expansion, sterility tests (Bact/Alert method), mycoplasma, and endotoxin tests were performed. Before cryopreservation, a EuPh sterility test was carried out. A cryopreserved DP unit was also used as control tube of the freezing–thawing process. This tube was then thawed for testing endotoxin and adventitious virus.

#### 3.1.4. Final Product (FP)

FP was generated by thawing two units of the DP, washing and adjustment to the final dose concentration, and conditioning for clinical use. In this final step, the presence of endotoxins, mycoplasma, and PhEu sterility testing was evaluated.

### 3.2. Identity of WJ-MSCs

**Figure 2:**
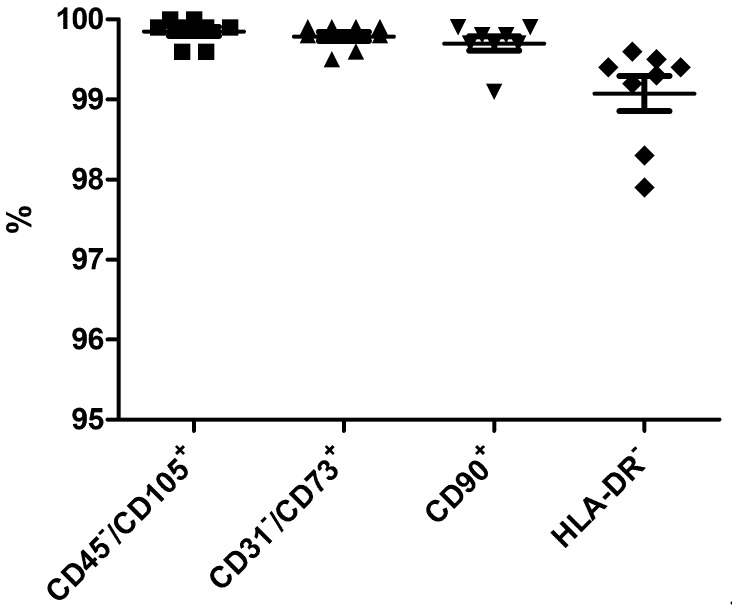
Immunophenotypic analyses of eight batches of clinical-grade WJ-MSC. The resulting values were always higher than 95%, in accordance with the established product specifications: 99.9 ± 0.2% CD45^−^/CD105^+^(■), 99.8 ± 0.2% CD31^−^/CD73^+^(▲), 99.7 ± 0.3% CD90^+^ (▼), 99.1 ± 0.6% HLA-DR^−^(♦).

Phenotypic profiling of WJ-MSCs was consistent with their mesenchymal identity, being positive for the expression of CD73, CD90, and CD105, and negative for CD31 and CD45, and HLA-DR (Figure 2 and Table 4). Viability of FP was 97.4 ± 0.5% (*n* = 8) with undetectable levels of endotoxins and mycoplasma DNA; and free of bacterial contamination and adventitious viruses in all cases. All karyotypes were normal, with 46 chromosomes (five of which were XY, two were XX, and one could not be determined due to low number of metaphase spreads).

### 3.3. Potency of WJ-MSCs

**Figure 3:**
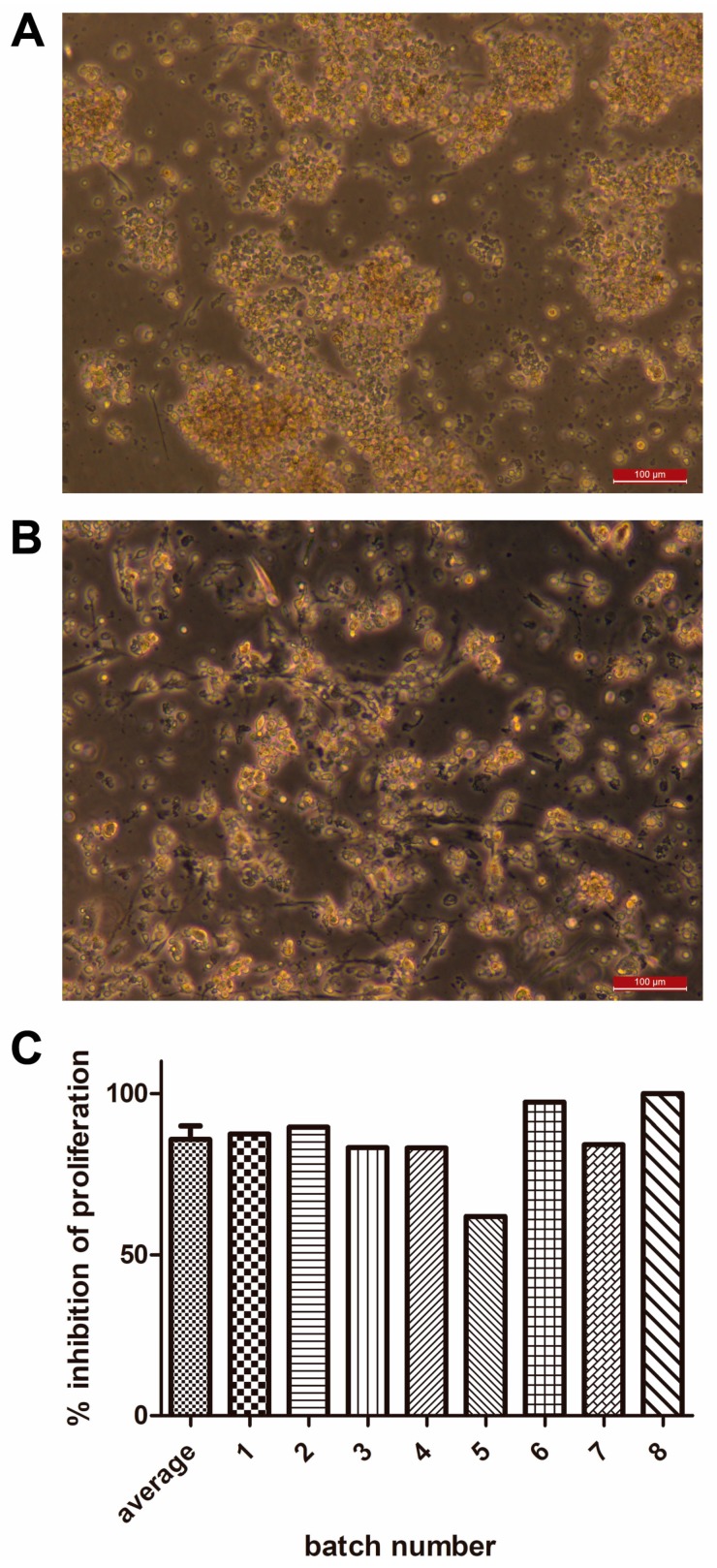
Results from lymphocyte proliferation assays (potency) in eight batches of clinical-grade WJ-MSC. Bright field microscopy images revealed clumping of peripheral blood MNC after 5 days in the of presence of 25 ng/mL PMA and 0.5 µM ionomycin (**A**), as opposed to same cells in co-culture with WJ-MSCs showing a dramatic decrease in the presence of such cell clumps (**B**). Values (in %) of the inhibition of the proliferation of activated lymphocytes are shown in (**C**). Scale bars = 100 µm.

The immunopotency of cells was determined in vitro by measuring the capacity of WJ-MSCs to inhibit the proliferation of polyclonally activated lymphocytes. In all cases, WJ-MSCs were able to inhibit proliferation 85.9 ± 10.9% (ranging from 61.9 to 100% inhibition, *n* = 8; Table 4). Additionally, microscopic pictures of stimulated PBMC were taken in the absence of WJ-MSCs (Figure 3A) when the formation of cell aggregates was observed and when WJ-MSCs were present and the consecutive reduction in cell clumps (Figure 3B), which correlates to immunopotency results shown in Figure 3C.

### 3.4. Risk Analysis

**Figure 4:**
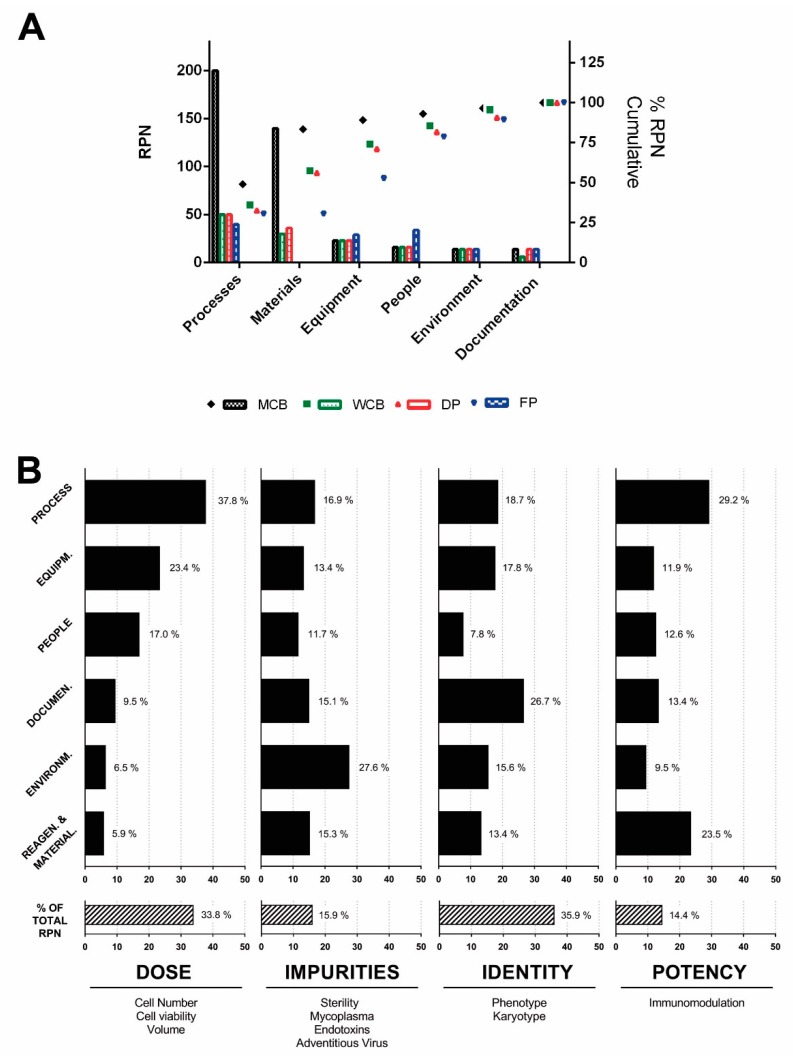
Risk analysis. (**A**) A Pareto chart was generated and plotted according to the classification and the failure group. (**B**) Graphical representation of the impact of risks from each failure group on critical quality attributes. The graphical view of the risk analyses for each of the failure groups provided invaluable assistance to focus efforts in the mitigation of critical risks affecting the specifications of MSCs in the final product. This methodology permits to streamline the identification of the weakest points of the process that deserve the implementation of further in-process controls or to preserve existing ones. MCB: Master cell bank; WCB: Working cell bank; DP: Drug product; FP: Final product; RPN: Risk priority number.

Risk analysis of failure groups was performed and plotted with its RPN for each step of the bioprocess, with the establishment of the MCB associated to the highest risk level regarding processes and materials (Figure 4A). Main risks were associated with endotoxins and adventitious virus due to two main sources: (a) Materials used and (b) open-system steps in the process. Indeed, isolation of the umbilical cord was performed during a nonsterile process, such as birth delivery. These microbiological parameters were not evaluated at this bank level. Importantly, while in the next steps, adventitious virus always happened to be negative, a variability was observed in the endotoxin level across the cell banking level. Furthermore, another high-risk level was presented by the raw material used for this process. Therefore, it was decided to perform endotoxin testing also at the MCB level to enhance the OOS detectability in the early process stages.

Moreover, an FMEA risk analysis of the complete manufacturing process was performed, taking into account potential risks that could impact on the critical quality attribute (CQA) (Table 3 and Tables S5–S8). Regarding CQA of the final product, the design of the bioprocess has a major impact on the dose and immunopotency of WJ-MSCs (Tables S5 and S8), whereas environmental factors impacted mainly on impurities (Table S6), and reagents and materials used could mostly affect WJ-MSCs’ immunomodulation capacity (Figure 4B and Table S8). Interestingly, no high vulnerabilities were found for failure modes in the determination of identity (Table S7). With the identification of potential risks, measures of mitigation concluded in the reduction of RPN overall values. However, new measures of risk mitigation can be further applied to increase risk mitigation.

## 4. Discussion

The interest in using MSCs for clinical applications has increased over the last few years. To date, nearly 1000 registered clinical studies over the world have involved the use of this cell type (www.clinicaltrials.gov; search terms: “mesenchymal” AND “stem” AND “cell”; April 2019), which can be explained because of their unique properties, namely: Multilineage differentiation potential, tropism to injured tissues, immunoregulatory capacity, and the release of growth factors and cytokines in response to environmental cues [15]. Altogether, these make MSCs highly attractive for treating a variety of diseases ranging from immune disorders to tissue regeneration [16].

Particularly, WJ-MSCs offer multiple advantages over the use of bone marrow-derived MSCs, such as the use of non-invasive procedures for tissue collection, standardization of donor age, and lack of somatic mutations, amongst others. Therefore, the umbilical cord emerges as an alternative source of MSCs, involving straightforward and pain-free collection [17]. Indeed, WJ-MSCs can be extensively expanded in culture and survive freeze/thaw cycles, thus making them suitable for the generation of cell banks of advanced therapy medicinal products available in an off-the-shelf format [15,18]. Moreover, WJ-MSCs have similar immunomodulation properties to BM-MSCs [19,20], which supports the use of WJ-MSCs in graft versus host disease (GvHD) and in the treatment of other immunological disorders along with BM-MSCs [21]. Interestingly, the use of MSCs sourced from different tissues may result in different effects in GvHD treatments, as they hold potentially different characteristics. Indeed, Grégoire and collaborators observed in vitro and in vivo, using a humanized mouse model, that MSCs show different effects on immune cells according to their source [22]. Therefore, standardization of a robust potency assay for MSCs is urgent to make possible the comparison of results from different laboratories and ensure that in vitro functionality for GMP-manufactured products prior to administration correlates to their clinical effect.

Although MSCs have been studied for more than 40 years, their translation into GMP-compliant processes is far from being straightforward and requires a deep analysis of several parameters to ensure quality, efficacy, and safety of the final product [23]. In this context, concepts of QbD offer a systematic approach that combines scientific knowledge and risk assessment that can assist developers and regulators in the understanding of parameters in bioprocesses that directly affect CQA of the final product. In the last few decades, QbD has become an essential tool in the successful development of pharmaceutical products, and consequently, regulatory organisms worldwide have adopted this concept in their guidelines. Herein we reported the integration of risk management and bioprocess design in the production of WJ-MSCs with immunomodulatory properties, providing main risks that may affect the quality of the final product so the bioprocess can be improved by mitigating risks. This approach contributes to increasing consistency and decreasing OOS.

In this work, we presented the successful production of eight batches of WJ-MSC for clinical use, all of them in compliance with GMP guidelines. Our bioprocess design received manufacturing authorization by the competent regulatory authority, including the described potency assay based on immunomodulation potential. Although we are aware of other methods to assess immunopotency, it is not clear whether any of them can represent the actual mechanisms of action of the drug substance in vivo [24]. Regulatory environments for drug manufacturing have imposed more stringent requirements in terms of quality controls to ensure safety and efficacy throughout the life cycle of the products. This is particularly challenging in the field of cell therapy because the active ingredient is made of living cells, and current analytical techniques lack standardization across laboratories [25]. We propose a sound starting point to quantify cell functionality beyond surrogate markers of potency that is usually limited to cell viability or mesenchymal phenotype identity. Interestingly, the quantification of the immunomodulation potential of WJ-MSCs as described here prospectively covers a wide range of possible mechanisms of action in vivo having the paracrine mode of action as a common characteristic. In this regard, our next objective is the evaluation of the proposed potency assay by analyzing whether clinical results somehow correlate with the quantitative attribute derived of the immunomodulation test.

## 5. Conclusions

The bioprocess design showed here and the implementation of a robust immunomodulation potency assay has enabled us to streamline the production of clinical-grade WJ-MSCs under pharmaceutical standards. Moreover, the application of FMEA risk management tool has provided an in-depth fundamental understanding of the process weaknesses allowing the implementation of the corresponding corrective actions to ensure the CQA in the final product as well as consistency batch-to-batch.

## Figures and Tables

**Table 1:** Acceptance criteria of starting material.

Sample	Requirement	Informative
**Maternal Blood**	Negative for: HBsAb, HIV I/II, syphilis (TPPA), Chagas, HBcAb, HCV, anti-HTLV I/II, NAT (HCV-HIV, HBV), anti-Toxo IgM, anti-EBV VCA IgM, anti-CMV IgM	Anti-Toxo IgG, anti- EBV VCA IgG, anti-CMV IgG
**Cord Blood**	HBsAb, HIV I/II, syphilis (TPPA), Chagas, HBcAb, HCV, anti-HTLV I/II, NAT (HCV-HIV, HBV)	N/A
**Umbilical Cord Fragment**	≥7 g, ≤ 80 h from birth	N/A

NAT: Nucleic acids test; HIV: Human immunodeficiency virus; CMV: Cytomegalovirus; EBV: Epstein–Barr virus; HBV: Hepatitis B virus; HCV: Hepatitis C virus; TPPA: Treponema pallidum particle agglutination assay; Toxo IgM: Toxoplasma immunoglobulin M; HBcAb: Hepatitis B core antibody; HBsAb: Hepatitis B surface antibody; HTLV: human T-cell leukemia–lymphoma virus; VCA: Viral capsid antibody.

**Table 2:** Release criteria for drug product.

Critical Quality Attribute	Value
**Dose**	1 × 10^7^ ± 20%
**Viability**	≥70%
**Phenotype**	CD45^-^/CD105^+^ ≥ 95%
	CD31^-^/CD73^+^ ≥ 95% CD90^+^ ≥ 95% HLA-DR ^1^
**Sterility**	Sterile
***Mycoplasma***	Negative
**Endotoxin**	≤1EU/mL
**Adventitious Viruses**	Negative
**Immunomodulation**	Positive

^1^ HLA-DR for informative purposes only.

**Table 3:** Risk analysis.

Critical Quality Attribute	Potential Failure Risk
**Dose**	Seeding density lower than the critical minimum
	Cell viability lower than 70%
	Slow cell growth
	Cell loss along the manufacturing process (washing and concentration steps)
**Impurities**	Contamination of the culture
	Endotoxins out of range
	Positive result for virus adventitious
**Identity**	Phenotype markers out of range
	Karyotype alteration
**Potency**	Failure in the immunomodulation test

**Table 4:** Critical quality attributes for each of the 8 batches of drug product.

	1	2	3	4	5	6	7	8
**Dose (×10^7^)**	2.5	2.4	2.3	2.3	2.5	2.4	2.5	2.8
**Num. doses**	18	6	24	14	16	20	39	41
**Viability (%)**	97.3	94.4	95.9	98.2	97.1	97.1	97.1	97.6
**CD45^−^/CD105^+^**	99.9	99.6	100	99.6	99.9	99.9	99.9	100
**CD31^−^/CD73^+^**	99.9	99.5	99.8	99.6	99.8	99.9	99.9	99.9
**CD90^+^**	99.8	99.1	99.9	99.7	99.9	99.7	99.8	99.7
**HLA-DR^-^**	99.4	98.3	99.2	97.9	99.4	99.3	99.6	99.5
**Karyotype**	46X, X	46X, X	n.d.	46X, Y	46X, Y	46X, Y	46X, Y	46X, Y
**Sterility**	-	-	-	-	-	-	-	-
***Mycoplasma***	-	-	-	-	-	-	-	-
**Endotoxin**	≤1EU/mL	≤1EU/mL	≤1EU/mL	≤1EU/mL	≤1EU/mL	≤1EU/mL	≤1EU/mL	≤1EU/mL
**Adventitious viruses**	-	-	-	-	-	-	-	-
**Immunomodulation**	87.5	89.6	83.3	83.2	61.9	97.3	84.2	100

n.d.: Not determined; -: Negative.

## Supplementary Materials

The following are available online at https://www.mdpi.com/2073-4409/8/5/484/s1, Table S1: Severity rating for the FMEA analysis, Table S2: Occurrence rating for the FMEA analysis, Table S3: Detection rating for the FMEA analysis, Table S4: Potential failures in the production of WJ-MSCs. Table S5. Potential failures regarding product’s dose. Complete Failure Mode and Effects Analysis for cell number, cell viability and volume. Table S6. Potential failures regarding product’s impurities. Complete Failure Mode and Effects Analysis for sterility, Mycoplasma, endotoxins, and adventitious viruses. Table S7. Potential failures regarding product’s identity. Complete Failure Mode and Effects Analysis for phenotype and karyotype. Table S8. Potential failures regarding the immunopotency of WJ-MSC. Complete Failure Mode and Effects Analysis for immunomodulation potential.
